# Development and validation of a multivariable prediction model of central venous catheter-tip colonization in a cohort of five randomized trials

**DOI:** 10.1186/s13054-022-04078-x

**Published:** 2022-07-07

**Authors:** Jeanne Iachkine, Niccolò Buetti, Harm-Jan de Grooth, Anaïs R. Briant, Olivier Mimoz, Bruno Mégarbane, Jean-Paul Mira, Stéphane Ruckly, Bertrand Souweine, Damien du Cheyron, Leonard A. Mermel, Jean-François Timsit, Jean-Jacques Parienti

**Affiliations:** 1 Department of Clinical Research and Biostatistics, Caen University Hospital and Caen Normandy University, Caen, France; 2 INSERM U1311 DynaMicURe, Caen Normandy University, Caen, France; 3 Infection Control Program and World Health Organization Collaborating Center On Patient Safety, Hospitals and Faculty of Medicine, University of Geneva, Geneva, Switzerland; 4 Department of Intensive Care, Amsterdam UMC Location Vrije Universiteit Amsterdam, De Boelelaan 1117, Amsterdam, The Netherlands; 5 Department of Biostatistics and Clinical Research, Caen University Hospital, Avenue de La Côte de Nacre, 30001, F-14000 Caen, CS France; 6 Inserm U1070, Poitiers University, Poitiers University Hospital, 86021 Poitiers, France; 7 Medical and Toxicological Intensive Care Unit, Lariboisière Hospital, AP-HP, INSERM UMRS-1144, Paris University, Paris, France; 8 Medical ICU, Cochin Hospital, AP-HP, 27 rue du Faubourg Saint-Jacques, 75014 Paris, France; 9 ICURESEARCH, Paris, France; 10 Intensive Care Unit, Gabriel-Montpied University Hospital, Clermont-Ferrand, France; 11 Department of Medical Intensive Care, Caen University Hospital, 14000 Caen, France; 12 Department of Epidemiology and Infection Prevention, Lifespan Hospital System, Providence, Rhode Island USA; 13 Department of Medicine, Warren Alpert Medical School of Brown University, Providence, Rhode Island USA; 14 Medical and Infectious Diseases ICU (MI2), Bichat Hospital, AP-HP, IAME, INSERM, University of Paris, U1137 Paris, France

**Keywords:** Catheter-tip colonization, Catheter-related infection, Intensive care unit, Central Venous catheters, Predictive score

## Abstract

**Background:**

The majority of central venous catheters (CVC) removed in the ICU are not colonized, including when a catheter-related infection (CRI) is suspected. We developed and validated a predictive score to reduce unnecessary CVC removal.

**Methods:**

We conducted a retrospective cohort study from five multicenter randomized controlled trials with systematic catheter-tip culture of consecutive CVCs. Colonization was defined as growth of ≥10^3^ colony-forming units per mL. Risk factors for colonization were identified in the training cohort (CATHEDIA and 3SITES trials; 3899 CVCs of which 575 (15%) were colonized) through multivariable analyses. After internal validation in 500 bootstrapped samples, the CVC-OUT score was computed by attaching points to the robust (> 50% of the bootstraps) risk factors. External validation was performed in the testing cohort (CLEAN, DRESSING2 and ELVIS trials; 6848 CVCs, of which 588 (9%) were colonized).

**Results:**

In the training cohort, obesity (1 point), diabetes (1 point), type of CVC (dialysis catheter, 1 point), anatomical insertion site (jugular, 4 points; femoral 5 points), rank of the catheter (second or subsequent, 1 point) and catheterization duration (≥ 5 days, 2 points) were significantly and independently associated with colonization . Area under the ROC curve (AUC) for the CVC-OUT score was 0.69, 95% confidence interval (CI) [0.67–0.72]. In the testing cohort, AUC for the CVC-OUT score was 0.60, 95% CI [0.58–0.62]. Among 1,469 CVCs removed for suspected CRI in the overall population, 1200 (82%) were not colonized. The negative predictive value (NPV) of a CVC-OUT score < 6 points was 94%, 95% CI [93%–95%].

**Conclusion:**

The CVC-OUT score had a moderate ability to discriminate catheter-tip colonization, but the high NPV may contribute to reduce unnecessary CVCs removal. Preference of the subclavian site is the strongest and only modifiable risk factor that reduces the likelihood of catheter-tip colonization and consequently the risk of CRI.

*Clinical Trials Registration*: NCT00277888, NCT01479153, NCT01629550, NCT01189682, NCT00875069.

**Supplementary Information:**

The online version contains supplementary material available at 10.1186/s13054-022-04078-x.

## Introduction

Central venous catheters (CVC), namely CVCs used to administer drugs and catheters used to deliver renal-replacement therapies (DC), are essential in the care of critically ill patients. In Europe in 2017, CVC use was 70.1 per 100 patient-days [1]. Up to two thirds of patients admitted in Intensive Care Units (ICU) are exposed to at least one CVC [2].

Unfortunately, their routine use is responsible for serious complications [3]. Among these, the most life threatening is catheter-related bloodstream infection (CRBSI). Although its incidence is decreasing over time with a current rate of 0.2 to 5/1000 catheters-days [1, 4–6], CRBSI is associated with mortality, prolonged ICU stays and higher hospitals costs [7–9]. While guidelines exist for the diagnosis and management, the strict definition of CRBSI relies on catheter-tip culture after catheter removal, or blood cultures, which may delay the diagnosis [10, 11]. When a CRBSI occurs, urgent removal of the CVC is the cornerstone of the management strategy. Obtaining positive blood cultures may be difficult under broad-spectrum antibiotics, which led to the catheter-related infection (CRI) definition (non-bacteremic clinical sepsis plus catheter-tip colonization or CRBSI). There is no consensus on how to define and act on suspected CRI, a very frequent situation in the ICU [10, 11].

Despite the importance of this topic, very few studies have investigated the optimal strategy in case of suspected CRI. Evidence for the safety of watchful waiting remains scarce. A randomized controlled trial (RCT) by Rijnders et al. [12] compared CVC removal left at the physician’s discretion (SOC) with watchful waiting amongst hemodynamically stable patients without proven bacteremia, no insertion site infection and no intravascular foreign body. The authors found no significant difference between the two groups in terms of CRBSI incidence and ICU mortality, but the sample size was small (*n* = 64). Of note, significantly fewer catheters were removed in the “watchful waiting” arm (62% reduction compared to the SOC arm, *p* < 0.01). Similar results were observed in a prospective observational study by Lorente et al. [13]. On the other hand, the absence of focus eradication (i.e., CVC removal) is clearly associated with mortality in the setting of CRBSI [14–16]. Consequently, the decision to remove the CVC is generally left at the physician’s judgement and experience. Of note, the fear of CRBSI on its own may trigger CVC replacement in a new anatomical site. However, systematic CVC replacement also exposes patients to potential harm [17]. These include mechanical complications when inserting a CVC in a new site. Moreover, the proportion of catheters that are not colonized after removal for suspected infection is significant in the series, up to 91% [18]. Developing tools to identify CVCs with a low likelihood of colonization could help reduce this proportion of premature CVC replacement. The objective of this study was to develop and validate a score capable of assessing the risk of CRI.

## Material and methods

### Study design

This report complies with the TRIPOD statement [19].

In this post-hoc analysis, we used the databases from five multicenter RCTs, for which data had been collected prospectively. We constructed the training cohort, used to develop the risk score, by merging 3SITES and CATHEDIA [3, 20]. Both studies investigated the impact of the anatomical insertion site on mechanical and intravascular catheter complications. The trials included in this training cohort were chosen by convenience because of the timing of availability of 3SITES and CATHEDIA databases. Subsequently, we used data from ELVIS, CLEAN and DRESSING2, which investigated different prevention strategies on the incidence of CRI [21–23], as testing cohort for external validation.

### Study patients and catheters

Inclusion criteria slightly differed between the studies and are detailed in Additional File 1. All patients were adults admitted to ICU and requiring catheterization through a new venipuncture. Data for more than one catheter per patient could be recorded in the five studies (we therefore define the rank of the catheter as equal to one for the first catheter inserted during the ICU stay and > 1 for the second and subsequent catheters).

Regarding the current study, focus was made on CVCs and DCs, with exclusion of arterial catheters. Catheters with missing data concerning the colonization at removal were excluded.

### Outcome and definitions

The principal outcome was colonization, defined as growth of ≥ 10^3^ colony-forming units per milliliter from the catheter-tip culture according to Brun-Buisson quantitative technique [24]. Interventions were blinded to microbiologists who processed catheter cultures. CRI was defined as the association of colonization with local (pus or inflammation at the insertion site) or systemic signs such as fever (body temperature ≥ 38.5 °C) or hypothermia (body temperature ≤ 36.5 °C) in the absence of any other identified infection focus, corresponding to the CRI-1 and CRI-2 subgroups proposed by the European Centre for Disease prevention and Control (ECDC) [25]. Suspicion of CRI was based on clinical judgement, and the physicians caring for each patient independently made the decision to remove the CVC in this situation. CRBSI was defined as a combination of at least one positive peripheral blood culture sampled immediately before or within 48 h after catheter removal, colonization of the catheter tip with the same microorganism or a differential time to positivity of blood cultures ≥ 2 h, in the absence of any other infectious focus explaining the positive blood culture result.

### Statistical analysis

No computation of sample size was performed a priori. Baseline characteristics of the patients and catheters included in the study were described using numbers (percentages) for categorical variables and median [inter-quartile range] for quantitative variables.

All the RCTs included in this work prospectively recorded demographics, clinical and microbiological data with a particular attention to risk factors associated with CVCs infection (Additional File 1). The statistical analysis plan had four steps that are detailed in the Additional File 2. The number of observations with missing data was low, and the imputation strategies are described in the Additional File 2.

Briefly, we identified independent risk factors of catheter-tip colonization from appropriate univariable and multivariable regression models in the training cohort [26]. We checked the absence of interaction between each risk factor and “CRI suspected” as the reason for CVC removal. Then, we assessed internal validity by resampling methods to retain only robust risk factors, and to estimate their coefficients to correct for over optimism [27]. Based on the strength of their association with the risk of catheter-tip colonization, we derived a 0–12 points score (called “CVC-OUT”) with a higher value signifying higher catheter-tip colonization probability [28]. Finally, the external validity of the CVC-OUT score was tested in the testing cohort. In both cohorts, the discrimination of the score was assessed using Receiving Operator Characteristics (ROC) curves and their c-index. The fitness was tested by the Hosmer and Lemeshow test. All analyses were performed with SAS software V9.4 (SAS Institute, NC, Cary) and R software (R Foundation for Statistical Computing, Vienna, Austria).

## Results

### Patients and catheters

**Fig. 1:**
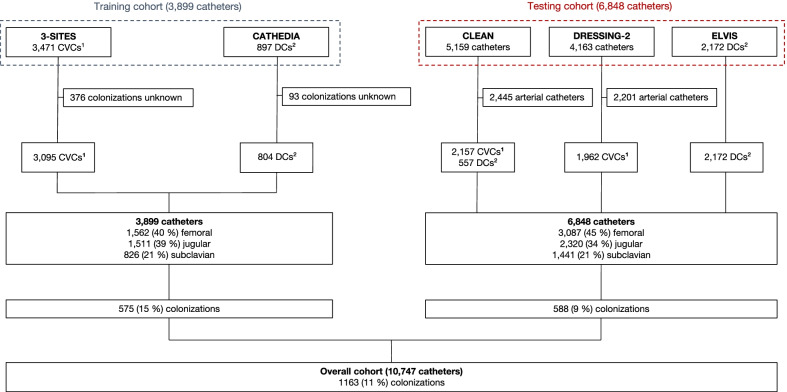
Flowchart. ^1^CVC, Central Venous Catheter; ^2^DC, Dialysis Catheter

In the training cohort, 3471 CVCs in the 3SITES study and 897 DCs in the CATHEDIA study were available. Among these 4368 catheters, 469 were excluded due to missing data regarding the colonization status, representing a total of 3899 catheters included in the study, of which 3095 (79%) were CVCs and 804 (21%) dialysis catheters (Fig. 1). Baseline characteristics of patients and catheters are presented in Table 1. Most patients were male (65%), with a median age of 65 years. 845 patients (22%) had a body mass index (BMI) > 30 kg/m^2^, and 833 (21%) had diabetes. Data concerning BMI were missing for 225 (6%) patients, and data concerning a past medical history of diabetes were missing for 331 (9%) patients. 2289 patients (59%) received vasopressors and 3127 (80%) required mechanical ventilation. Data concerning vasopressors infusion at admission were missing for 336 (9%) patients. At insertion, 1,405 patients (36%) received anticoagulants and 2300 (59%) received antibiotic therapy. Forty percent (*n* = 1562), 39% (*n* = 1511) and 22% (*n* = 826) of catheters were inserted in the femoral, jugular and subclavian vein, respectively. Antiseptic used for skin disinfection was mostly iodine-povidone (58%). A total of 546 catheters (14%) were second or subsequent catheters. Median dwell time was 5 days.

**Table 1:** Patients and catheters characteristics in the training cohort and in the testing cohort

	Training cohort (*n* = 3899)^1^		Testing cohort (*n* = 6848)^1^	
	Colonization	No colonization	Colonization	No colonization
Total, *n*^1^ (%)	575 (15)	3324 (85)	588 (9)	6260 (91)
*Patients*^4^				
Male, *n* (%)	372 (65)	2,167 (65)	359 (61)	4023 (64)
Age, median [IQR]	67 [55–77]	64 [52–75]	64 [55–74]	64.0 [54–74]
BMI^2^, median [IQR]	27 [23–31]	26 [22–29]	28 [23–33]	26 [23–31]
Obesity^5^, *n* (%)	165 (29)	680 (21)	207 (35)	1663 (27)
Diabetes, *n* (%)	162 (28)	671 (20)	58 (10)	570 (9)
SAPS2^3^, median [IQR]	57 [45–70]	55 [42–70]	57 [43–73]	57 [44–73]
Mechanical ventilation, *n* (%)	442 (77)	2685 (81)	439 (75)	4584 (73)
Antibiotic therapy at admission, *n* (%)	331 (58)	1969 (59)	357 (61)	4056 (65)
Vasopressors at admission, *n* (%)	327 (57)	1962 (59)	285 (49)	3471 (56)
*Catheters*				
At insertion				
Type of catheter, *n* (%)				
Central venous catheter	371 (65)	2724 (82)	320 (54)	3799 (61)
Dialysis catheter	204 (36)	600 (18)	268 (46)	2461 (39)
First catheter inserted, *n* (%)	459 (80)	2894 (87)	246 (42)	2959 (47)
Skin disinfection with chlorhexidine, *n* (%)	132 (23)*	1280 (39)*	207 (35)	3862 (62)
Skin disinfection with alcohol-based povidone-iodine, *n* (%)	419 (73)*	1851 (56)*	381 (65)	2398 (38)
*Insertion site, n (%)*				
Subclavian	34 (6)	792 (24)	85 (14)	1356 (22)
Jugular	240 (42)	1271 (38)	246 (42)	2074 (33)
Femoral	301 (52)	1261 (38)	257 (44)	2830 (45)
*At removal*				
Time to removal > 5 days, *n* (%)	354 (62)	1528 (46)	380 (65)	2956 (47)
Removal for suspected catheter-related infection, *n* (%)	120 (21)	477 (14)	149 (25)	723 (12)

^1^n is for number of catheters; ^2^BMI, body mass index; ^3^SAPS2, Simplified Acute Physiology Score 2; ^4^Patients with several CVCs inserted are counted several time for baseline characteristics. The ratio CVC/patients was 1.2 in the training cohort and 1.4 in the testing cohort; ^5^obesity was defined as body mass index ≥ 30 kg/m^2^

*Total may not be 100% since use of other antiseptics was allowed

In the testing cohort (Fig. 1), 6848 catheters were enrolled, of which 4119 (60%) CVCs and 2729 (40%) DCs. Patients were mostly male (64%), with a median age of 64 years. At admission, 5023 (73%) required mechanical ventilation, and 3756 (55%) received vasopressors. 4413 (64%) patients received antibiotics. 45% (*n* = 3087), 34% (*n* = 2320) and 21% (*n* = 1441) of catheters were inserted in the femoral, jugular and subclavian vein respectively.

The distribution of important covariates in the training and testing cohorts is available in Table 1.

In the training cohort, 575 (15%, 95% confidence interval (CI) [14–16]) catheters were colonized. Among 597 catheters removed for suspected CRI, 477 (80%, 95% CI [77–83]) were culture-negative. In the testing cohort, 588 (9%, 95% CI [8, 9]) catheters were colonized. Among 872 catheters removed for suspected CRI, 723 (83%, 95% CI [80–85]) were culture-negative.

The overall cohort represented 10,747 catheters, of which 1163 (11%, 95% CI [10, 11]) were colonized. Among 1469 catheters removed for suspected CRI, 1200 (82%, 95% CI [80–84]) were culture-negative.

### Predictors of outcome

On univariable analysis, nine factors were significantly associated with colonization: age > 60 years, obesity, diabetes, mechanical ventilation, use of anticoagulants, site of insertion (femoral and jugular versus subclavian), type of catheter (DC versus CVC), successful insertion at first attempt and dwell time. The first catheter inserted was significantly less likely to be colonized (Table 2).

**Table 2:** Univariable and multivariable analyses

	Univariable analysis (*n* = 3899)			Multivariable analysis (*n* = 3899)		
	OR^1^	CI^2^ 95%	*p*-value	Adjusted OR^1^	CI^2^ 95%	*p*-value
Male	0.98	[0.81–1.19]	0.86			
Age > 60 years	1.39	[1.15–1.68]	** < 0.001**	1.23	[1.01–1.49]	**0.04**
Obesity^3^	1.56	[1.28–1.92]	** < 0.001**	1.37	[1.11–1.69]	**0.004**
Immunosuppression^4^	0.94	[0.75–1.19]	0.63			
Diabetes	1.55	[1.26–1.91]	** < 0.001**	1.32	[1.06–1.64]	**0.012**
SAPS2^5^	1	[1.00–1.01]	0.2381			
Mechanical ventilation at insertion	0.79	[0.64–0.97]	**0.028**	0.79	[0.63–1.00]	**0.046**
Antibiotic therapy at insertion	0.94	[0.78–1.12]	0.47			
Vasopressors at insertion	0.92	[0.76–1.1]	0.35			
Anticoagulation at insertion^6^	1.28	[1.07–1.53]	**0.008**	–	–	–
Dialysis catheter	2.5	[2.05–3.03]	** < 0.001**	1.82	[1.48–2.23]	** < 0.001**
*Insertion site*						
Subclavian	1	–	–	1.00	–	–
Jugular	4.21	[2.96–5.99]	** < 0.001**	3.75	[2.61–5.4]	** < 0.001**
Femoral	5.32	[3.76–7.53]	** < 0.001**	4.41	[3.06–6.34]	** < 0.001**
First catheter inserted	0.59	[0.47–0.73]	** < 0.001**	0.70	[0.55–0.89]	**0.004**
Successful insertion at first attempt	1.24	[1.03–1.51]	**0.026**	–	–	–
Mechanical complication at insertion^7^	0.9	[0.65–1.27]	0.56			
Dwell time > 5 days	1.88	[1.57–2.26]	** < 0.001**	1.93	[1.59–2.33]	** < 0.001**

Bold values indicated statistically significant

^1^OR, odds ratio

^2^CI, confidence interval

^**3**^obesity was defined as body mass index ≥ 30 kg/m^2^

^4^immunosuppression is defined as a combination of primary immune disorder, treatment by immunosuppressants, active solid or hematological malignancy or HIV infection

^5^SAPS2, simplified acute physiology score 2

^6^anticoagulation refers to administration of heparin through the catheter

^7^mechanical complications include arterial punction, hematoma, pneumothorax, hemothorax and malposition

None of the risk factor interacted significantly with “suspected CRI” as the reason for CVC removal. No evidence of collinearity was observed (Additional file 3: Table S1), and all ten covariates were introduced in the multivariable model. After backward and forward selection, age > 60 years, obesity, diabetes, mechanical ventilation, site insertion, type of catheter (DC versus CVC), rank of the catheter (first versus subsequent) and dwell time > 5 days were significantly associated with colonization. In contrast, use of anticoagulants and successful insertion at first attempt were no longer associated with colonization on multivariable analysis (Table 2). The sensitivity analysis consisting in repeating the multivariable analysis in a five-time imputed dataset was similar (Additional file 4: Table S2).

**Fig. 2:**
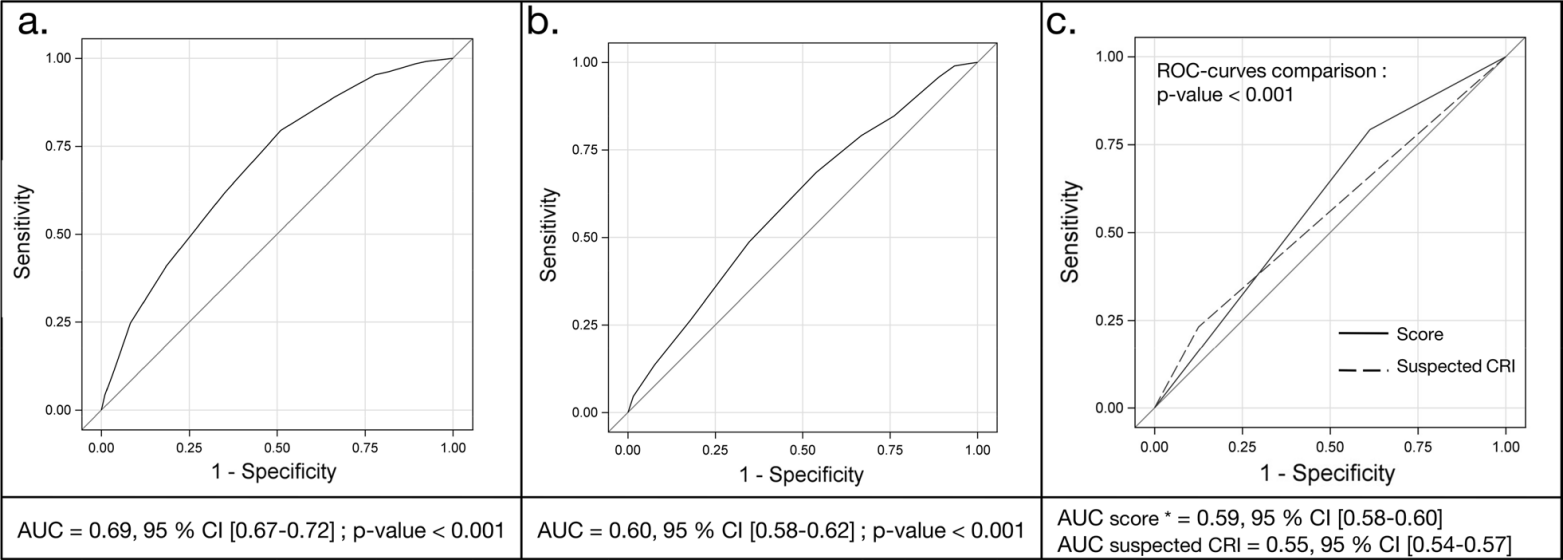
ROC curves for the simplified based-points score in the training, testing, and overall cohorts. *Threshold ≥ 6 points (“high risk group”) versus < 6 points (“low risk group”) **a**. shows the receiving operator comparison (ROC) curve in the training cohort, **b**. shows the ROC curve in the testing cohort and **c**. shows the comparison between a CVC-OUT score ≥ 6 points and the suspicion of catheter-related infection (CRI) in the overall cohort. AUC, area under curve; CI, confidence interval; CRI, catheter-related infection

Based on 500-time repetitions of the multivariable model in bootstrap samples and sub-samples, obesity, diabetes, site of insertion, type of catheter, rank of the catheter and dwell time were considered as robust risk factors for colonization (Additional file 5: Table S3). To derive the CVC-OUT score as a simple points-based score, points were attached to the predictors according to their bootstrap coefficients. The method used to calculate the points system is detailed in Additional file 6. Obesity, diabetes and rank of the catheter > 1 accounted for 1 point. Type of the catheter (DC) and dwell time > 5 days accounted for 2 points. Insertion site accounted for 4 or 5 points respectively in case of jugular or femoral insertion. The score ranged from 0 (predicted probability for colonization = 2.6%, 95% CI [1.9–3.4]) to 12 points (predicted probability for colonization = 50.6%, 95% CI [44.6–56.4]). The ROC curve is presented in Fig. 2. Area under curve (AUC) was 0.69, 95% CI [0.67–0.72], and the Hosmer and Lemeshow goodness of fit test denoted satisfying adequation to the data (*p* = 0.64). Using the ROC curve and the zenith of the Youden index to determine the threshold, a CVC-OUT score ≥ 6 points defined the “high risk” group and predicted colonization with a sensitivity of 79.5% (95% CI [75.9–82.7]) and a specificity of 49.0% (95% CI [47.2–50.7]). The positive predictive value (PPV) was 21.2% (95% CI [20.3–22.1]) and the negative predictive value (NPV) was 93.2% (95% CI [92.1–94.2]) (Additional file 7: Table S4). Comparison of the observed percentages of colonized catheters and the CVC-OUT score predicted probability in the training cohort is displayed in Additional file 8: Figure S1.

### External validation

In the testing cohort, the CVC-OUT score ranked from 0, predicting a colonization probability of 3.4% (95% CI [2.6–4.4]), to 12, predicting a colonization probability of 17.2% (95% CI [14.4–20.5]). AUC was 0.60, 95% CI [0.58–0.62], *p* < 0.001 (Fig. 2). The sensitivity was 79.1% (95% CI [75.6–82.3]) and the specificity was 33.2% (95% CI [32.1–34.4]). The use of CVC-OUT score < 6 points allowed excluding the diagnosis of colonization with a NPV of 94.4% (95% CI [93.5–95.2]) Additional file 7: Table S4. Comparison of the observed percentages of colonized catheters and the CVC-OUT score predicted probability in the testing cohort is displayed in Additional file 8: Figure S1.

### Predictive performance in the overall cohort

**Fig. 3:**
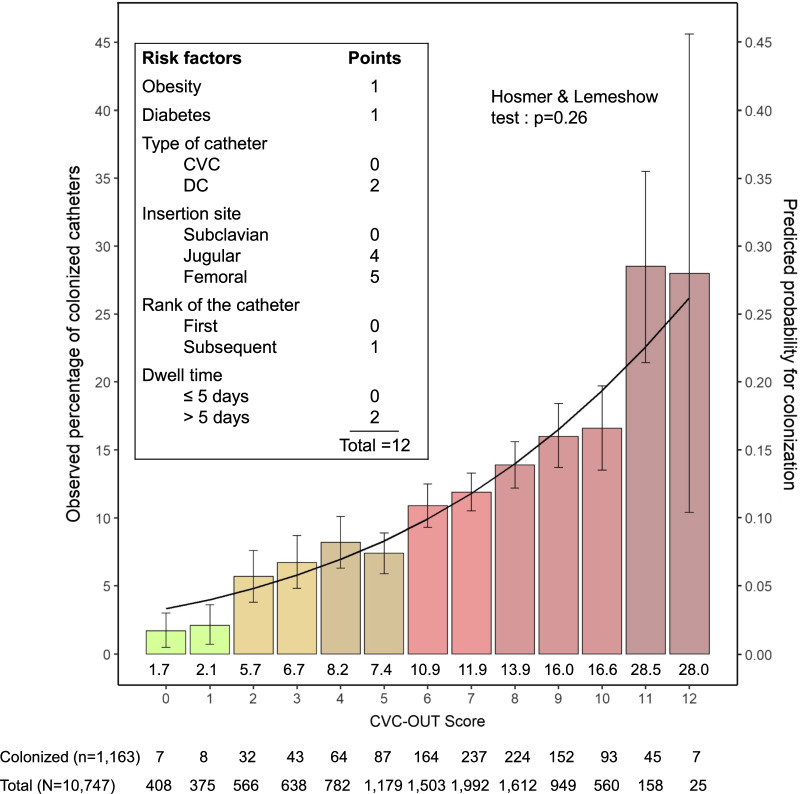
Comparison of the observed percentage of colonized catheters and the score-predicted probability for colonization by points total in the overall cohort (*n* = 10,747)

The CVC-OUT score in the overall cohort ranked from 0 (predicted probability 3.3%, 95% CI [2.7–4.0]) to 12 points (predicted probability 26.2%, 95% CI [23.3–29.2]). Figure 3 displays the comparison between observed percentages of colonized CVCs and score-predicted probability for colonization by CVC-OUT score in the overall cohort.

AUC for the CVC-OUT score in the overall cohort was 0.63, 95% CI [0.61–0.64]. As shown in Table 3, the negative predictive value (NPV) of a CVC-OUT score < 6 points was 93.9%, 95% CI [93.2%–94.5%]. A score ≥ 6, predicted colonization better than the presence of a suspicion of CRI (p < 0.001 for the comparison of AUCs) (Fig. 2).

**Table 3:** Contingency table for a CVC-OUT score ≥ 6 in the overall cohort (*n* = 10,747)

	Colonized catheters, *n* (%)	Culture-negative catheters, *n* (%)	Total, *n* (%)
CVC-OUT score ≥ 6, n (%)	922 (8.6)	5877 (54.7)	6799 (63.3)
CVC-OUT score < 6, *n* (%)	241 (2.2)	3707 (34.5)	3948 (36.7)

Sensitivity = 79.3%, 95% confidence interval (CI) [76.8–81.6]

Specificity = 38.7%, 95% CI [37.7–39.7]

Positive predictive value = 13.6%, 95% CI [13.2–14.0]

Negative predictive value = 93.9%, 95% CI [93.2–94.5]

Among catheters removed for suspected CRI (*n* = 1469), removing only those with a score ≥ 6 could avoid 402 unnecessary removals and reinsertions (28.6%, 95% CI [26.3–30.9]) at the cost of 59 colonized catheters left in situ (4.0%, 95% CI [3.0–5.0]). Among the catheters removed for suspected CRI in the overall cohort, AUC for the simplified points score was 0.59, 95% CI [0.55–0.62] (Additional file 9: Figure S2).

### Sensitivity analysis

After exclusion of catheters colonized with coagulase-negative *Staphylococci* (CoNS), 357 out of 3,861 (10%, 95% CI [9–11]) catheters in the training cohort and 345 out of 6396 (5%, 95% CI [5, 6]) catheters in the testing cohort were colonized. Age, obesity, diabetes, insertion site (jugular and femoral vs subclavian), type of catheter (CVC vs. DC) and dwell time > 5 days were significantly associated with colonization and proved robust after the bootstrap selection (Additional file 10: Tables S4 and S5).

## Discussion

Using the high-quality individual-patient data from five large RCTs investigating the prevention of CRI in the ICU, we developed and validated a score with a moderate discrimination but a good negative predictive value to predict catheter-tip colonization. The presence of a low CVC-OUT score may encourage physicians to adopt a watchful waiting strategy that is safer while remaining CVCs in place in case of suspicion of CRI.

The points system makes the score easy to use at the bedside and allows considering the weight of each risk factor. In our study, the femoral and the jugular veins, respectively 5 and 4 points were the factors most strongly associated with colonization. Interestingly, anatomical insertion site is modifiable. A prospective study by Timsit et al. identified femoral and internal jugular sites of insertion and dwell time ≥ 5 days as independent risk factors for catheter-tip colonization [29]. In our study, dwell time was also associated with colonization with the odds increasing almost twofold when the catheter was left in place more than five days. This finding suggests that it is appropriate to favor subclavian access when the expected duration of catheterization is long, as supported by recent guidelines [30]. Increasing the rate of subclavian CVCs inserted in the ICU will not only reduce the risk of intravascular complications [3] but will also provide reassurance that any potential infection symptoms occurring during the ICU stay are less likely to be catheter-related. Of note, the subclavian route is associated with a higher risk of mechanical complications, including pneumothorax [3]. The choice of the insertion site must therefore remain based on an individual assessment of the benefit-risk ratio.

The proportion of femoral CVCs was high in our study, about 43%. Two reasons could explain this: (i) DCs represented 21% and 40% of the training and testing cohorts, respectively, limiting the choice of insertion site to the jugular and femoral veins. The CATHEDIA RCT did not support to avoid femoral central venous access for DCs, except amongst obese patients [20]. Second, the 3SITES RCT investigated the anatomic site for CVC insertion in a 1:1:1 ratio, so a third of participants had femoral CVCs [3].

Consistent with our observation, a recent study showed that BMI ≥ 40 kg/m^2^ was associated with catheter colonization [31]. This could be explained by more frequent dressing disruptions, and by insertion made more difficult by the loss of anatomical landmarks and deeper vessels, thus favoring more manipulations [32].

Most studies focus on CRI or CRBSI and few have investigated the risk factors for colonization [29, 31, 33]. The clinical relevance of colonization (regardless of the presence of CR-1 or CR-2) is debated as a surrogate endpoint for CRBSI [34, 35] but it represents the first step to CRI [36]. The clinical context of CRI suspicion (fever or local signs of inflammation in presence of a CVC) provides more importance to catheter-tip colonization, as it naturally excludes asymptomatic patients. Of note, CRI is a moderate to good surrogate endpoint for CRBSI [34, 35]. Predicting colonization risk in this context, or more importantly its low likelihood with high negative predictive value could support the “watchful waiting” strategy, and help reduce the prevalence of non-colonized catheters being removed.

As we focused on colonization, we performed a sensitivity analysis by excluding catheters for which the tip culture was positive to CoNS only. Although CoNS are frequently associated with CRBSI [1, 37], in the absence of positive blood cultures, identification of CoNS on catheter-tip culture is difficult to interpret. The continuum between contamination and colonization with pathogenic potential is blurred, as CoNS tends to be less virulent [38], and may be less likely to cause local or systemic symptoms [39].

Our study has several limitations. We investigated colonization, which is not a clinical outcome such as CRI. Therefore, our findings do not support CVC removal in asymptomatic patients who have a high CVC-OUT score without another indication for removing the CVC. Because of its retrospective design, unmeasured covariates and missing data may cause residual confounding even though data collection was prospective in all five RCTs, and performed by trained investigators. Data regarding antibiotic therapy at catheter removal were not available, and it may have affected the results of catheter-tip cultures. Indeed, a study by Souweine et al. raised awareness that antibiotics administrated at the time of catheter removal may interfere with microbiological diagnosis [40]. All five studies were conducted before the current French Guidelines, and practice has evolved since then. It has now been clearly demonstrated that skin disinfection with alcoholic chlorhexidine is more effective than povidone iodine to prevent CRI [6, 21, 22]. The difference in the prevalence of colonization in the training and testing cohorts may be partially explained by a higher prevalence of skin disinfection with 2% chlorhexidine alcohol in the testing cohort. We chose not to adjust the multivariable model on the type of skin antiseptic used, because using alcoholic 2% chlorhexidine is now recommended in most guidelines [10, 11, 30]. This may have artificially decreased the discrimination of the CVC-OUT score by omitting the skin antisepsis or dressings used in the trials. For the same reason, we did not adjust the multivariable model on the use of ultrasonography guidance at catheter insertion, even though a recent study showed that the use of ultrasonography might favor asepsis breaches and thus increase the risk of CRI [41]. Of note, the use of evidence-based prevention strategies that reduce the risk of CRI will tend to increase our negative predictive value by decreasing the incidence of catheter-tip colonization. Our study included both: CVCs and DCs. They differ in design, use (with more manipulations for DCs) and insertion site [42]. The subclavian vein, while preferred to reduce the CVC infection rate, is discouraged for DCs insertion because of the risk of central-vein stenosis [43]. Finally, the potential contribution of the immunocompromised state of those patients needing dialysis or more frequent manipulations of the central line may explain why DC were at higher risk of colonization, compared to CVCs. Because the interventions differed within the studies, there could be an interaction between the intervention and the outcome. Nevertheless, studies included in the training cohort (3SITES and CATHEDIA) both investigated the impact of insertion site on catheter-related complications, with randomization of the insertion site, thus limiting heterogeneity within the training cohort. Finally, the five multicenter RCTs were conducted exclusively in France, which potentially limits the generalizability of our results.

In conclusion, the CVC-OUT score showed a moderate ability to discriminate catheter-tip colonization but a high negative predictive value and allowed to apprehend the weight of each risk factor. Preference of the subclavian site was the strongest and only modifiable factor influencing the risk of CVC-tip colonization. Of note, the use of real-time ultrasound decreases the risk of mechanical complications in subclavian insertions as compared to landmark technique, without being associated with an increased risk of infection [41]. Whether the use of CVC-OUT is effective to reduce unnecessary CVC removal in the clinical context of suspected CRI requires further prospective evaluations.

## Supplementary Information

**Additional file 1** **Annex 1: **Detailed presentation of the randomized controlled trials included in the study: Text describing inclusion/exclusion criteria and interventions for the five trials included in the study.

**Additional file 2** **Annex 2:** Detailed Statistical Methods: Text describing the statistical analysis.

**Additional file 3 Table S1: **Variance Inflation Factors for the covariates included in the multivariable model in the principal analysis.

**Additional file 4 Table S2: **Univariable and multivariable analyses in the five time imputed dataset as part of a sensitivity analysis.

**Additional file 5** **Table S3: **Relative frequency (%) with which each candidate predictor was selected in 500 bootstrap samples from the training cohort and correspondence between the bootstrapped coefficients for the robust risk factors and attached points in the simplified points-based score.

**Additional file 6** **Annex 3: **Method for the determination of the attached points in the points-based system. Description of the process of derivating points from the coefficients.

**Additional file 7 Table S4: **Contingency tables for the CVC-OUT score in the training cohort and in the testing cohort.

**Additional file 8** **Figure S1: **Comparison of the observed percentage of colonized catheters and the score-predicted probability for colonization by points total in the training and testing cohorts panel **a**. displays the training cohort and panel **b**. displays the testing cohort.

**Additional file 9** **Figure S2:** ROC curve for the simplified points-based score in the sub-group of catheters removed for suspected catheter-related infection.

**Additional file 10** **Table S4: **Univariable and multivariable analyses after exclusion of the catheters colonized with Coagulase negative Staphylococci as part of a sensitivity analysis. Table S5: Relative frequency (%) with which each candidate predictor was selected in 500 bootstrap samples from the training cohort and bootstrapped coefficients for the robust risk factors after exclusion of the catheters colonized with Coagulase negative Staphylococci as part of a sensitivity analysis.

## Data Availability

The datasets used and analyzed during the current study are available from the corresponding author on reasonable request.

## Abbreviations

CVC: Central venous catheter
DC: Catheters used for renal replacement therapy
ICU: Intensive care unit
CRBSI: Catheter-related bloodstream infection
CRI: Catheter-related infection
RCT: Randomized controlled trial
ECDC: European Centre for Disease Prevention and Control
ROC curve: Receiver operating characteristics curve
CI: Confidence interval
OR: Odds ratio
AUC: Area under curve
PPV: Positive predictive value
NPV: Negative predictive value
CoNS: Coagulase-negative *Staphylococci*
