# Supplementary material for: Development and validation of a multivariable prediction model of central venous catheter-tip colonization in a cohort of five randomized trials
Source: Crit Care. 2022 Jul 7;26:205. doi: 10.1186/s13054-022-04078-x (PMC9261073; doi:10.1186/s13054-022-04078-x)
Supplement: Supplementary file 1 — Additional file 1 Annex 1: Detailed presentation of the randomized controlled trials included in the study: Text describing inclusion/exclusion criteria and interventions for the five trials included in the study. [file 13054_2022_4078_MOESM1_ESM.pdf]

Annex 1: Detailed presentation of the randomized controlled trials included in the study

A. Training cohort

The 3SITES [1] study compared subclavian, jugular and femoral access towards major central catheter-related complication, defined as the composite of catheter related blood stream infection and symptomatic deep vein thrombosis.

Recruitment was conducted from December 2011 to June 2014. Patients were recruited from 10 French ICUs, in 4 university-affiliated hospitals and 5 general hospitals and were followed until death or Intensive Care Unit (ICU) discharge.

Inclusion criteria were adult patients (≥ 18 years) admitted to ICU, requiring catheterization with a central venous catheter through a new venipuncture, and considered to be suitable for insertion in at least two sites(among internal jugular veins, subclavian veins and femoral veins).

Patients were randomized in a 1:1:1 randomization scheme if all the three venous sites were suitable for catheter placement. If one of the three sites was not suitable on both the left and right sides of the body, the catheterization site was assigned in a 1:1 randomization scheme for the other two sites (two-choice scheme).If only one site was suitable, the catheterization procedure was not included in the study. Randomization was stratified according to ICU and according to the use of antibiotic therapy.

The primary outcome was the incidence of major catheter related complications from the time of insertion to 48 hours after catheter removal. Major complications were defined as the composite of catheter-related bloodstream infection (CRBSI) and symptomatic deep-vein thrombosis, whichever occurred first.

The CATHEDIA [2] study assessed the effect of the insertion site (jugular vs femoral) on the time to colonization at removal of dialysis catheters.

Recruitment was conducted between May 2004 and May 2007. Patients were recruited from 9 university-affiliated hospitals and 3 general hospitals and were followed until death or Intensive Care Unit (ICU) discharge.

Eligible patients were included critically-ill adult patients requiring insertion of a dialysis catheter by a senior physician for expected support with renal replacement therapy (RRT). Exclusion criteria were contra-indication to insertion in one site, body mass index (BMI) > 45 kg/m², coagulopathy, local skin infection, profound volume overload, presence of arteriovenous fistula and thoracic life threatening condition.

Patients were randomized in a 1:1 ratio between internal-jugular catheterization and femoral catheterization. Randomization was stratified by center and type of RRT.

The primary outcome was the time to colonization at catheter removal.

B. Testing cohort

The ELVIS [3] study assessed ethanol lock efficiency on the prevention of major catheter-related infections in ICU patients requiring short-term dialysis catheters.

Recruitment was conducted respectively June 2009 and December 2011, in 16 ICUs in 7 university-affiliated hospitals and 1 general hospital. Patients were followed until death or 48 hours after ICU discharge.

Eligible patients were adults requiring insertion of a non-tunneled and non-antimicrobial-impregnated dialysis catheter, with an expected duration of use longer than 48 hours. Exclusion criteria were known intolerance to ethanol and pregnancy.

Patients were randomly assigned in a 1:1 ratio to either a 60 % ethanol lock or a 0.9 % saline lock.

The primary outcome was the number of major catheter related infection (CRI) episodes per catheter. Major CRI was defined as either catheter-related sepsis without CRBSI or CRBSI.

The CLEAN [4] study compared skin disinfection using chlorhexidine-alcohol versus povidone iodine-alcohol regarding the incidence of catheter-related infection.

Recruitment was conducted between October 2012 and February 2014, in 11 French ICUs in 5university-affiliated hospitals and 1 general hospital. Patients were followed until death or 48 hours after ICU discharge.

Eligible patients were consecutive critically ill adult patients requiring arterial, dialysis or central venous catheters for at least 48 hours. Exclusion criteria were contraindication to any trial antiseptic, high risk of death within the 48 first hours or need to use an antimicrobial-coated catheter.

Patients were randomly assigned in a 1:1:1:1 ratio to one of the four treatment groups (chlorhexidine alcohol or povidone iodine alcohol, with administration preceded or not by skin scrubbing with an antiseptic detergent).

The primary outcome was the incidence of CRI.

Finally, the DRESSING2 [5] study compared three types of transparent dressings regarding the catheter colonization rate and the major catheter related infection rate.

Recruitementwas conducted from May 2010 to July 2011, in 12 ICUs in 7 university-affiliated hospitals and 4 general hospitals. Patients were followed until death or 48 hours after ICU discharge.

Eligible patients were consecutive ICU patients expected to require intravascular catheterization for at least 48 hours. Exclusion criterion was known allergy to chlorhexidine or to transparent dressings.

Patients were randomly assigned to one of the three dressings (chlorhexidine gel impregnated dressing or highly adhesive dressing or standard transparent dressing). Randomization was stratified by ICU.

The primary outcome was either the catheter colonization rate for highly adhesive dressings versus standard dressings or major CRI rate for chlorhexidine impregnated dressings versus non chlorhexidine dressings. Major CRI was defined as CRBSI or catheter-related sepsis without CRBSI.

C. Trials Procedures

Insertion took place in the ICU, and was performed by junior or senior physicians depending on the studies. Maximal sterile barrier precautions including surgical hand antisepsis, use of large sterile drapes, and use of sterile gowns and gloves were used. Skin disinfection used either alcoholic povidone-iodine or alcoholic chlorhexidine. Catheterization was achieved by means of the Seldinger technique, with the use of anatomical landmarks or ultra-sonographic guidance. Catheters were not used for routine blood sampling. Catheters were removed when no longer required, or when a new access was required, as decided independently by the physicians caring for each

patient. Patients discharged from the ICU with the catheter in place had blood cultures drawn from the catheter and from a peripheral vein simultaneously to take account of the absence of catheter-tip culture.

References:

1. Parienti J-J, Mongardon N, Mégarbane B, Mira J-P, Kalfon P, Gros A, Marqué S, Thuong M, Pottier V, Ramakers M, Savary B, Seguin A, Valette X, Terzi N, Sauneuf B, Cattoir V, Mermel LA, du Cheyron D (2015) Intravascular Complications of Central Venous Catheterization by Insertion Site. N Engl J Med 373(13):1220-1229. https://www.nejm.org/doi/10.1056/NEJMoa1500964

2. Parienti J-J, Thirion M, Mégarbane B, Souweine B, Ouchikhe A, Polito A, Forel J-M, Marqué S, Misset B, Airapetian N, Daurel C, Mira J-P, Ramakers M, du Cheyron D, Le Coutour X, Daubin C, Charbonneau P (2008) Femoral vs Jugular Venous Catheterization and Risk of Nosocomial Events in Adults Requiring Acute Renal Replacement Therapy: A Randomized Controlled Trial. JAMA 299:2413. https://doi.org/10.1001/jama.299.20.2413

3. Souweine DB, Lautrette DA, Gruson D, Canet E, Klouche K, Argaud L, Bohe J, garrouste-Orgeas M, Mariat C, Vincent F, Cayot S, Cointault O, Lepape A, Guelon D, Darmon M, Vesin A, Caillot N, Schwebel C, Boyer A, Azoulay E, Bouadma L, Timsit J-F (2015) Ethanol Lock and Risk of Hemodialysis Catheter Infection in Critically Ill Patients: A Randomized Controlled Trial. Am J Respir Crit Care Med 191(9):1024-1032. https://doi.org/10.1164/rccm.201408-1431OC

4. Mimoz O, Lucet J-C, Kerforne T, Pascal J, Souweine B, Goudet V, Mercat A, Bouadma L, Lasocki S, Alfandari S, Friggeri A, Wallet F, Allou N, Ruckly S, Balayn D, Lepape A, Timsit J-F (2015) Skin antisepsis with chlorhexidine–alcohol versus povidone iodine–alcohol, with and without skin scrubbing, for prevention of intravascular-catheter-related infection (CLEAN): an open-label, multicentre, randomised, controlled, two-by-two factorial trial. The Lancet 386:2069–2077. https://doi.org/10.1016/S0140-6736(15)00244-5

5. Timsit J-F, Mimoz O, Mourvillier B, Souweine B, Garrouste-Orgeas M, Alfandari S, Plantefeve G, Bronchard R, Troche G, Gauzit R, Antona M, Canet E, Bohe J, Lepape A, Vesin A, Arrault X, Schwebel C, Adrie C, Zahar J-R, Ruckly S, Trounegros C, Lucet J-C (2012) Randomized Controlled Trial of Chlorhexidine Dressing and Highly Adhesive Dressing for Preventing Catheter-related Infections in Critically Ill Adults. 186(12):1272-1278. https://doi.org/10.1164/rccm.201206-1038OC
