# Supplementary material for: Development and validation of a multivariable prediction model of central venous catheter-tip colonization in a cohort of five randomized trials
Source: Crit Care. 2022 Jul 7;26:205. doi: 10.1186/s13054-022-04078-x (PMC9261073; doi:10.1186/s13054-022-04078-x)
Supplement: Supplementary file 2 — Additional file 2 Annex 2: Detailed Statistical Methods: Text describing the statistical analysis. [file 13054_2022_4078_MOESM2_ESM.pdf]

Annex 2: Detailed methods

Demographic data such as age and sex category were recorded at admission. The Simplified Acute Physiology Score (SAPS2) was calculated within the first 24 hours following Intensive Care Unit (ICU) admission and was used to assess the severity of disease. Past medical history, especially presence of immunosuppression, obesity, diabetes, was recorded for every patient. Data concerning medication at the time of catheterization was recorded regarding antibiotic therapy and vasopressors (epinephrine, norepinephrine or dobutamine) in the five trials. Type of catheter, namely central venous catheters used for drugs administration (CVC) vs catheters used for renal-replacement therapy (DC), site of insertion, rank of the catheter, catheterization success at first attempt and occurrence of mechanical complications (pneumothorax, arterial puncture, hematoma or hemothorax) were recorded at insertion for every catheter. Dates of insertion and removal were used to calculate the dwell time.

Numeric variables were considered as continuous when the log-linearity relation with the outcome was verified, as for Simplified Acute Physiology Score 2 (SAPS2). SAPS2 was calculated within the first 24 hours following ICU admission. Body mass index (BMI) was categorized as ≥ 30 defining obesity (World Health organization definition). Time to removal was dichotomized as "long" for catheters left in place for more than five days, and "short" for catheters left in place for five days or less, as a median-cut. Age was categorized as ≥ 60 years and < 60 years (World Health Organization definition for "elderly").

Assuming a missing at random pattern, single imputation of missing data regarding key variables was performed to allow exploitation of all observations. BMI was imputed as predicted by linear regression of age and sex, and for qualitative variables, a missing data was considered as the absence of the event. Covariates with data missing for > 20 % of the observations were not selected to be part of the analyses. Sensitivity analyses with multiple imputations were performed using five-time imputed dataset, using the fully conditional specification method.

First, we investigated potential risk factors for colonization in the training cohort. Variables considered as clinically relevant were assessed in univariate analysis. We used a generalized linear model with binomial distribution fit with generalized estimating equation (GEE) to account for the dependence of observations from catheters drawn from the same patients [1]. All variables associated with colonization (p-value <0.20) in univariate analysis were selected for inclusion in the multivariate analysis. We assessed collinearity between these variables by calculating the variance inflation factor (VIF). The VIF did not exceed 5 for any variable, denoting the absence of collinearity. The same model was used for multivariate analysis. We used backward and forward selection procedures among

qualified variables to select independent risk factors associated with colonization, with the threshold of $p < 0.05$ to remain in the model. This threshold was chosen to decrease the number of variables finally included in the predictive score, to make it easier and more practical to use. In addition, we conducted a sensitivity analysis consisting in repeating the multivariate analysis in a five-time imputed dataset, to compare the qualified variables regarding the imputation method (single imputation versus multiple imputation).

Second, we assessed the internal validity (stability and consistency) of our model, by conducting the same multivariate model in 500 bootstrap samples drawn from the training cohort, which allowed to assess the distribution of an indicator variable specifying the statistical significance ($p < 0.05$) for each predictor variable. This procedure was performed in the full-sized training cohort, and in random subsamples containing respectively 90%, 80% and 70% of the full-sized training cohort. We considered a risk factor robust when found significant in at least 50 % of the bootstrap samples and subsamples, as described by Mannan [2]. Odds-ratios (OR) and coefficients for the robust risk factors also were assessed using bootstrap samples.

The score was then computed using robust risk factors and their bootstrapped coefficients. In order to make it easier to use at patient's bedside, the score was developed as a simple points-based system, using the method of Sullivan and D'Agostino [3]. The constant for the points-based system (number of regression units corresponding to one point) was defined as the increase of risk associated with obesity. Predictors states associated with higher risk of colonization were assigned more points, meaning a higher point total represents a higher risk.

The predicting value of the simple points-based score was tested in the training cohort using the c-index, representing the area under receiver operating characteristic (ROC) curves (AUC) of the logistic model. Goodness of fit for the model was assessed through Hosmer and Lemeshow test. The Youden index was used to determine the threshold used to discriminate the "high risk group" and the "low risk group" (Youden index = sensitivity + specificity – 1).

External validation was performed in the testing cohort. The predictive value of the score was assessed in the testing cohort using ROC-curves and their c-index.

Finally, the training and the testing cohort were pooled. The overall cohort was used to compare the predictive value of the score, dichotomized as "high risk" or "low risk" as described before, to the presence of clinical suspicion of catheter related infection, by comparing the AUCs under the respective ROC curves. We also

calculated the observed percentage of colonized catheters and the score-predicted probability by points total in the overall cohort.

We conducted a sensitivity analysis by repeating the whole score development process after exclusion of the catheters colonized by coagulase-negative *Staphylococci*.

References:

1. Liang K-Y, Zeger SL (1986) Longitudinal data analysis using generalized linear models. Biometrika 73:13–22. https://doi.org/10.1093/biomet/73.1.13

2. Mannan H (2017) A Practical Application of a Simple Bootstrapping Method for Assessing Predictors Selected for Epidemiologic Risk Models Using Automated Variable Selection. International Journal of Statistics and Applications 7:239-249.

3. Sullivan LM, Massaro JM, D'Agostino RB (2004) Presentation of multivariate data for clinical use: The Framingham Study risk score functions. Statist Med 23:1631–1660. https://doi.org/10.1002/sim.1742
