# Supplementary material for: Development and validation of a multivariable prediction model of central venous catheter-tip colonization in a cohort of five randomized trials
Source: Crit Care. 2022 Jul 7;26:205. doi: 10.1186/s13054-022-04078-x (PMC9261073; doi:10.1186/s13054-022-04078-x)
Supplement: Supplementary file 3 — Additional file 3 Table S1: Variance Inflation Factors for the covariates included in the multivariable model in the principal analysis. [file 13054_2022_4078_MOESM3_ESM.pdf]

Supplemental Table 1: Variance Inflation Factors for the covariates included in the multivariate model in the principal analysis

**Covariates**	**Variance inflation factor**
Age > 60 years	1.04
Obesity	1.06
Diabetes	1.07
Mechanical ventilation	1.05
Anticoagulation	1.07
Dialysis catheter	1.15
Jugular insertion	1.79
Femoral insertion	1.89
First catheter inserted	1.05
Successful insertion at first attempt	1.05
Dwell time > 5 days	1.06
