# Supplementary material for: Development and validation of a multivariable prediction model of central venous catheter-tip colonization in a cohort of five randomized trials
Source: Crit Care. 2022 Jul 7;26:205. doi: 10.1186/s13054-022-04078-x (PMC9261073; doi:10.1186/s13054-022-04078-x)
Supplement: Supplementary file 4 — Additional file 4 Table S2: Univariable and multivariable analyses in the five time imputed dataset as part of a sensitivity analysis. [file 13054_2022_4078_MOESM4_ESM.pdf]

Supplemental Table 2: univariate and multivariate analyses in the five time imputed dataset as part of a sensitivity analysis

	Univariate analysis (n=3,899)			Multivariate analysis (n=3,899)		
	**OR[1]**	**95 % CI[2]**	**p**	**Adjusted OR[1]**	**95 % CI[2]**	**p**
**Male**	0.98	[0.81-1.19]	0.86			
**Age > 60 years**	1.39	[1.15-1.68]	**<0.001**	1.24	[1.02-1.50]	**0.034**
**Obesity**	1.49	[1.20-1.80]	**<0.001**	1.32	[1.06-1.64]	**0.012**
**Immunosuppression**	0.94	[0.75-1.19]	0.63			
**Diabetes**	1.53	[1.24-1.89]	**<0.001**	1.31	[1.05-1.62]	**0.016**
**SAPS2**	1.00	[1.00-1.01]	0.24			
**Mechanical ventilation at insertion**	0.79	[0.64-0.98]	**0.030**	0.79	[0.63-1.00]	**0.047**
**Antibiotic therapy at insertion**	0.94	[0.78-1.12]	0.47			
**Catecholamines at insertion**	0.85	[0.70-1.02]	**0.08**	-	-	-
**Anticoagulation at insertion**	1.28	[1.07-1.54]	**0.008**	-	-	-
**Dialysis catheter**	2.50	[2.05-3.03]	**<0.001**	1.81	[1.48-2.22]	**<0.001**
**Insertion site**						
**Subclavian**	1.00	-	-	1.00	-	-
**Jugular**	4.21	[2.96-5.99]	**<0.001**	3.74	[2.60-5.38]	**<0.001**
**Femoral**	5.32	[3.76-7.53]	**<0.001**	4.41	[3.07-6.36]	**<0.001**
**First catheter inserted**	0.59	[0.47-0.73]	**<0.001**	0.71	[0.55-0.90]	**0.005**
**Successful insertion at first attempt**	1.24	[1.03-1.51]	**0.026**	-	-	-
**Mechanical complication at insertion**	0.90	[0.65-1.27]	0.56			
**Dwell time > 5 days**	1.88	[1.57-2.26]	**<0.001**	1.93	[1.60-2.34]	**<0.001**

[1] OR, odds-ratio

[2] CI, confidence interval
