# Supplementary material for: Development and validation of a multivariable prediction model of central venous catheter-tip colonization in a cohort of five randomized trials
Source: Crit Care. 2022 Jul 7;26:205. doi: 10.1186/s13054-022-04078-x (PMC9261073; doi:10.1186/s13054-022-04078-x)
Supplement: Supplementary file 5 — Additional file 5 Table S3: Relative frequency (%) with which each candidate predictor was selected in 500 bootstrap samples from the training cohort and correspondence between the bootstrapped coefficients for the robust risk factors and attached points in the simplified points-based score. [file 13054_2022_4078_MOESM5_ESM.pdf]

Supplemental Table 3: Relative frequency (%) with which each candidate predictor was selected in 500 bootstrap samples from the training cohort and correspondence between the bootstrapped coefficients for the robust risk factors and attached points in the simplified points-based score

Risk factors	Full sample	90 % sample	80 % sample	70 % sample	β	Points
**Age > 60 years**	**55.4**	**58.6**	41.6	34.0	**-**	**-**
**Obesity**	**82.0**	**84.2**	**93.6**	**69.0**	0.33	1
**Diabetes**	**70.8**	**64.8**	**57.8**	**68.8**	0.32	1
**Mechanical ventilation**	**52.0**	45.2	19.0	26.6	-	-
**Dialysis catheter**	**100.0**	**100.0**	**100.0**	**100.0**	0.62	2
**Insertion site**						
**Subclavian**	**-**	**-**	**-**	-	0.00	0
**Jugular**	**100.0**	**100.0**	**100.0**	**100.0**	1.32	4
**Femoral**	**100.0**	**100.0**	**100.0**	**100.0**	1.50	5
**Rank of the catheter**						
**First**	**80.8**	**87.0**	**80.8**	**87.0**	-0.34	0
**Others**	**-**	**-**	**-**	**-**	**-**	1
**Dwell time > 5 days**	**100.0**	**100.0**	**99.4**	**99.2**	0.63	2

Bold frequency values are for risk factors retained in the robust model
