# Supplementary material for: Development and validation of a multivariable prediction model of central venous catheter-tip colonization in a cohort of five randomized trials
Source: Crit Care. 2022 Jul 7;26:205. doi: 10.1186/s13054-022-04078-x (PMC9261073; doi:10.1186/s13054-022-04078-x)
Supplement: Supplementary file 6 — Additional file 6 Annex 3: Method for the determination of the attached points in the points-based system. Description of the process of derivating points from the coefficients. [file 13054_2022_4078_MOESM6_ESM.pdf]

Annex 3: Method for the determination of the attached points in the points-based system [1]

Step 1 : Obtain multivariate regression coefficients

Risk factors	Bootstrapped coefficients (βi)
**Obesity**	0.33
**Diabetes**	0.32
**Subclavian insertion**	0.00
**Jugular insertion**	1.32
**Femoral insertion**	1.50
**Dialysis catheter**	0.62
**First catheter inserted**	-0.34
**Dwell time (> 5 days)**	0.63

Step 2 : definition of reference values, and referent risk profiles

Risk factors	Categories	Reference value (Wij)	Referent risk profile
**Obesity**	0	0.00	0.00
**Obesity**	1	1.00	0.00
**Diabetes**	0	0.00	0.00
**Diabetes**	1	1.00	0.00
**Jugular insertion***	0	0.00	0.00
**Jugular insertion***	1	1.00	0.00
**Femoral insertion***	0	0.00	0.00
**Femoral insertion***	1	1.00	0.00
**Dialysis catheter**	0	0.00	0.00
**Dialysis catheter**	1	1.00	0.00
**First catheter inserted**	0	0.00	1.00
**First catheter inserted**	1	1.00	1.00
**Dwell time (> 5 days)**	0	0.00	0.00
**Dwell time (> 5 days)**	1	1.00	0.00

* Site of insertion is coded as a dummy variable. Reference category is "subclavian insertion"

Step 3: Establish distance from base category

We now compute how far each category of each risk factor is from the base category in terms of regression unites using distance = $\beta_i(W_{ij} - W_{iREF})$

Risk factors	Categories	Reference value (W_{ij})	Referent risk profile(W_{iREF})	Bootstrapped coefficients (βi)	Distance from base category
**Obesity**	0	0.00	0.00	0.33	0.00
**Obesity**	1	1.00	0.00	0.33	0.33
**Diabetes**	0	0.00	0.00	0.32	0.00
**Diabetes**	1	1.00	0.00	0.32	0.32
**Jugular insertion***	0	0.00	0.00	1.32	0.00
**Jugular insertion***	1	1.00	0.00	1.32	1.32
**Femoral insertion***	0	0.00	0.00	1.50	0.00
**Femoral insertion***	1	1.00	0.00	1.50	1.50
**Dialysis catheter**	0	0.00	0.00	0.62	0.00
**Dialysis catheter**	1	1.00	0.00	0.62	0.62
**First catheter inserted**	0	0.00	1.00	-0.34	0.34
**First catheter inserted**	1	1.00	1.00	-0.34	0.00
**Dwell time (> 5 days)**	0	0.00	0.00	0.63	0.00
**Dwell time (> 5 days)**	1	1.00	0.00	0.63	0.63

* Site of insertion is coded as a dummy variable. Reference category is "subclavian insertion"

Step 5: Set the constant B

We now define the constant for the points system, namely the number of regression units that will correspond to one point. Here, we let B reflect the increase of risk associated with the presence of obesity (B=0,33)

Step 6: Determination of the points associated with each category of the risk factors

We now define the points associated with each category using $Points_{ij} = \frac{\beta_i(W_{ij} - W_{iREF})}{B}$

The result is rounded to the nearest integer to obtain the points.

Risk factors	Categories	Reference (Wij)	Referent risk profile (WiREF)	Bootstrapped coefficients (βi)	Distance from base category	Distance /B	Points
**Obesity**	0	0.00	0.00	0.33	0.00	0.00	0
**Obesity**	1	1.00	0.00	0.33	0.33	**1.00**	**1**
**Diabetes**	0	0.00	0.00	0.32	0.00	0.00	0
**Diabetes**	1	1.00	0.00	0.32	0.32	0.97	**1**
**Jugular insertion***	0	0.00	0.00	1.32	0.00	0.00	0
**Jugular insertion***	1	1.00	0.00	1.32	1.32	4.00	**4**
**Femoral insertion***	0	0.00	0.00	1.50	0.00	0.00	0
**Femoral insertion***	1	1.00	0.00	1.50	1.50	4.55	**5**
**Dialysis catheter**	0	0.00	0.00	0.62	0.00	0.00	0
**Dialysis catheter**	1	1.00	0.00	0.62	0.62	1.88	**2**
**First catheter inserted**	0	0.00	1.00	-0.34	0.34	1.03	**1**
**First catheter inserted**	1	1.00	1.00	-0.34	0.00	0.00	0
**Dwell time (> 5 days)**	0	0.00	0.00	0.63	0.00	0.00	0
**Dwell time (> 5 days)**	1	1.00	0.00	0.63	0.63	1.91	**2**

Reference:

1. Mannan H (2017) A Practical Application of a Simple Bootstrapping Method for Assessing Predictors Selected for Epidemiologic Risk Models Using Automated Variable Selection. International Journal of Statistics and Applications 7:239-249.
