# Supplementary material for: Development and validation of a multivariable prediction model of central venous catheter-tip colonization in a cohort of five randomized trials
Source: Crit Care. 2022 Jul 7;26:205. doi: 10.1186/s13054-022-04078-x (PMC9261073; doi:10.1186/s13054-022-04078-x)
Supplement: Supplementary file 7 — Additional file 7 Table S4: Contingency tables for the CVC-OUT score in the training cohort and in the testing cohort. [file 13054_2022_4078_MOESM7_ESM.pdf]

Supplemental Table 4: Contingency tables for the CVC-OUT score in the training cohort and in the testing cohort.

	**Training cohort (n=3,899)**			**Testing cohort (n=6,848)**		
	**Colonized catheters (n)**	**Sterile catheters (n)**	**Total (n)**	**Colonized catheters (n)**	**Sterile catheters (n)**	**Total (n)**
**CVC-OUT ≥ 6 (n)**	457	1,697	**2,154**	465	4,180	**4,645**
**CVC-OUT < 6 (n)**	118	1,627	**1,745**	123	2,080	**2,203**
**Total (n)**	**575**	**3,324**	**3,899**	**588**	**6,260**	**6,848**
