# Supplementary material for: Development and validation of a multivariable prediction model of central venous catheter-tip colonization in a cohort of five randomized trials
Source: Crit Care. 2022 Jul 7;26:205. doi: 10.1186/s13054-022-04078-x (PMC9261073; doi:10.1186/s13054-022-04078-x)

Supplemental Figure 1: Comparison of the observed percentage of colonized catheters and the score-predicted probability for colonization by points total in the training cohort (panel a.) and in the testing cohort (panel b.)

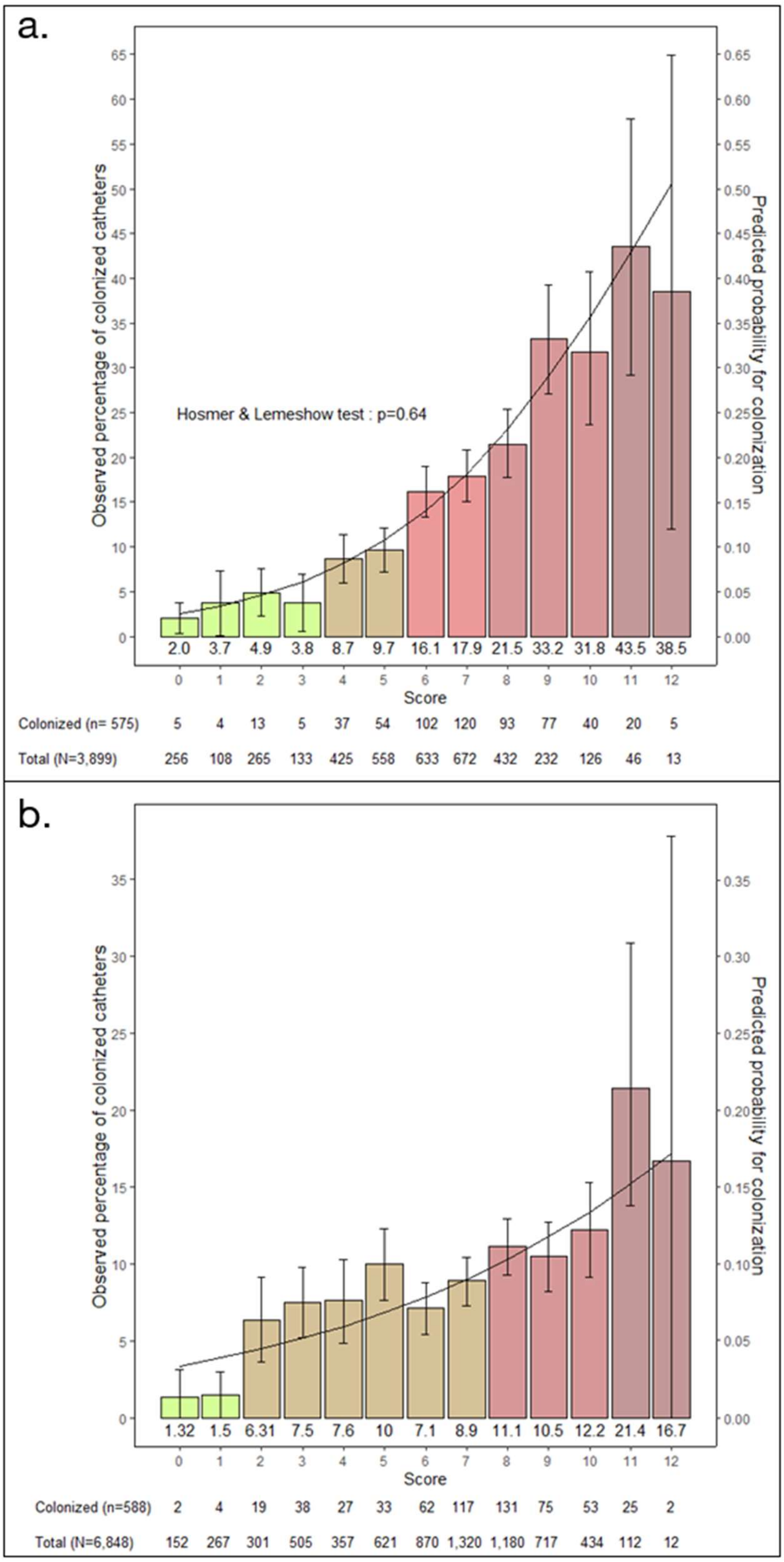

Supplement: Supplementary file 8 — Additional file 8 Figure S1: Comparison of the observed percentage of colonized catheters and the score-predicted probability for colonization by points total in the training and testing cohorts panel a. displays the training cohort and panel b. displays the testing cohort. [file 13054_2022_4078_MOESM8_ESM.pdf]
