# Supplementary material for: Development and validation of a multivariable prediction model of central venous catheter-tip colonization in a cohort of five randomized trials
Source: Crit Care. 2022 Jul 7;26:205. doi: 10.1186/s13054-022-04078-x (PMC9261073; doi:10.1186/s13054-022-04078-x)

Supplemental Figure 2: ROC curve for the simplified points-based score in the sub-group of catheters removed for suspected catheter related infection

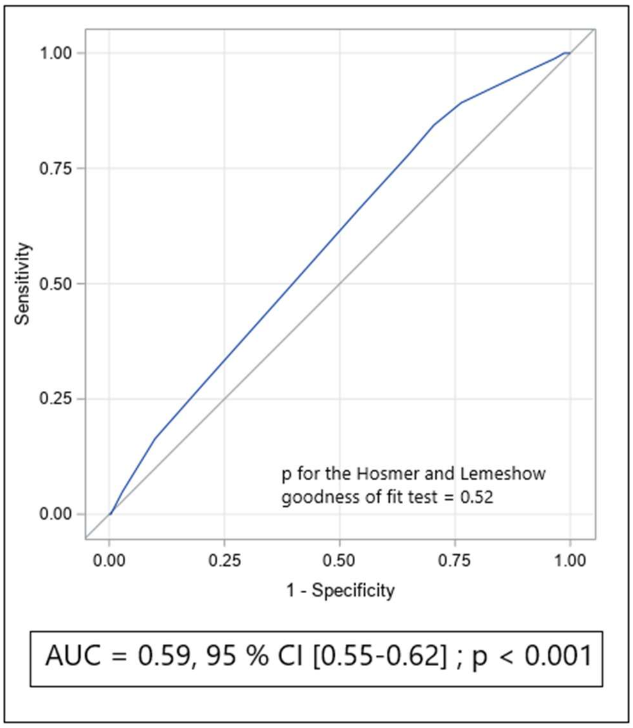

Supplement: Supplementary file 9 — Additional file 9 Figure S2: ROC curve for the simplified points-based score in the sub-group of catheters removed for suspected catheter-related infection. [file 13054_2022_4078_MOESM9_ESM.pdf]
