# Supplementary material for: Development and validation of a multivariable prediction model of central venous catheter-tip colonization in a cohort of five randomized trials
Source: Crit Care. 2022 Jul 7;26:205. doi: 10.1186/s13054-022-04078-x (PMC9261073; doi:10.1186/s13054-022-04078-x)
Supplement: Supplementary file 10 — Additional file 10 Table S4: Univariable and multivariable analyses after exclusion of the catheters colonized with Coagulase negative Staphylococci as part of a sensitivity analysis. Table S5: Relative frequency (%) with which each candidate predictor was selected in 500 bootstrap samples from the training cohort and bootstrapped coefficients for the robust risk factors after exclusion of the catheters colonized with Coagulase negative Staphylococci as part of a sensitivity analysis. [file 13054_2022_4078_MOESM10_ESM.pdf]

Supplemental Table 4: univariate and multivariate analyses after exclusion of the catheters colonized with Coagulase negative Staphylococci as part of a sensitivity analysis

	Univariate analysis (n= 3,681)			Multivariate analysis (n= 3,681)		
**Risk factors**	**OR[1]**	**95 % CI[2]**	**p-value**	**Adjusted OR[1]**	**95 % CI[2]**	**p-value**
**Male**	0.89	[0.70-1.12]	0.309			
**Age > 60 years**	1.52	[1.20-1.93]	**0.001**	1,38	[1.08-1.76]	**0.01**
**Obesity**	1.75	[1.37-2.25]	**<0.001**	1,5	[1.16-1.94]	**0.002**
**Immunosuppression**	0.96	[0.73-1.27]	0.783			
**Diabetes**	1.67	[1.30-2.14]	**<0.001**	1.4	[1.08-1.81]	**0.012**
**SAPS2***	1.00	[1.00-1.01]	0.111	-	-	-
**Mechanical ventilation**	0.9	[0.69-1.18]	0.449			
**Antibiotic therapy at insertion**	1.06	[0.85-1.33]	0.596			
**Parenteral nutrition**	0.74	[0.44-1.27]	0.281			
**Catecholamines**	0.94	[0.75-1.17]	0.568			
**Anticoagulation**	1.27	[1.02-1.59]	**0.032**	-	-	-
**Dialysis catheter**	2.13	[1.67-2.72]	**<0.001**	1.75	[1.34-2.27]	**0.002**
**Insertion site**						
**Subclavian insertion**	1.00	-	-	1.00	-	-
**Jugular insertion**	3.17	[2.034.94]	**<0.001**	3.03	[1.95-4.71]	**<0.001**
**Femoral insertion**	5.52	[3.605.50]	**<0.001**	5.11	[3.31-7.87]	**<0.001**
**First catheter inserted**	0.62	[0.47-0.82]	**0.001**	-	-	-
**Successful insertion at first attempt**	1.19	[0.94-1.50]	0.155	-	-	-
**Mechanical complication at insertion**	0.94	[0.62-1.41]	0.765			
**Dwell time > 5 days**	2.03	[1.62-2.54]	**<0.001**	1.95	[1.55-2.47]	**<0.001**

[1] OR, odds-ratio

[2] CI, confidence interval

*SAPS2, Simplified Acute Physiology Score 2

Supplemental Table 5: Relative frequency (%) with which each candidate predictor was selected in 500 bootstrap samples from the training cohort and bootstrapped coefficients for the robust risk factors after exclusion of the catheters colonized with Coagulase negative Staphylococci as part of a sensitivity analysis

Risk factors	Full sample	90 % sample	80 % sample	70 % sample	Bootstrapped coefficients
**Age > 60 years**	**73,8**	**63,2**	**70,6**	**83,4**	**0,31**
**Obesity**	**93,6**	**82,2**	**56**	**55**	**0,31**
**Diabetes**	**64,8**	**70,2**	**72,4**	**61,8**	**0,44**
**Insertion site**					
**Subclavian**	**-**	**-**	**-**	**-**	**-**
**Jugular**	**100**	**100**	**100**	**100**	**1,05**
**Femoral**	**100**	**100**	**100**	**100**	**1,6**
**Dialysis catheter**	**86,8**	**87**	**84,6**	**79,2**	**0,41**
**Dwell time > 5 days**	**100**	**100**	**100**	**99,6**	**0,71**
